# Tumour Suppressor Genes—One Hit Can Be Enough

**DOI:** 10.1371/journal.pbio.0020040

**Published:** 2004-02-17

**Authors:** Peter Hohenstein

## Abstract

A paper published in 1998 showed that loss of only one copy of the p53 tumor suppressor gene is sometimes enough to initiate carcinogenesis

For people who received their introduction to cancer genetics in college in the first half of the 1990s, everything looked simple and straightforward. It was the stuff you could explain to sincerely interested relatives who wanted to know what you were spending your time on. There were oncogenes and there were tumour suppressor genes. Oncogenes were overactive genes and proteins that somehow caused cancer because they were overactive; therefore, they were dominant. Tumour suppressor genes were genes that would normally prevent a tumour from happening and that needed to be inactivated for a tumour to start to form; both copies of a tumour suppressor gene had to be inactivated, and the mutation was recessive. If inactivation of these genes is a random process, it was understandable that people who inherit an inactivated copy of a tumour suppressor gene had a higher risk of developing the associated form(s) of cancer than people born with two normal copies, as postulated in Alfred Knudson's (1971) two-hit model. And, indeed, it was shown that in the tumours in these predisposed patients, the remaining wild-type copy of the tumour suppressor gene was lost, a process referred to as loss of heterozygosity.

For me, in 1998 things started to change. Venkatachalam et al. (1998) published a paper in the *EMBO Journal* describing a detailed study of tumours in mice lacking one copy of the p53 tumour suppressor gene (*Trp53*). This gene is known to be the most mutated gene in human cancer and its function to be central to many processes that are involved in the cellular prevention of cancer. Mice lacking both copies of this gene are for the most part viable, but succumb to cancer (mainly thymic lymphomas) at three to five months of age (Donehower et al. 1992). Mice born with one copy of the *Trp53* gene start to develop cancer at around nine months, and incidence increases with age.

In their study, Venkatachalam and colleagues analysed an impressive group of 217 *Trp53* ^+/−^ mice of controlled genetic background and followed the fate of the *Trp53* wild-type allele in the tumours. According to the two-hit model, it was expected that in these tumours this copy would have been lost or inactivated. However, this turned out not to be the case. Half of the tumours from mice younger than 18 months were found to have retained the wild-type copy of *Trp53*, a number that increased to 85% in mice older than 18 months. In two tumours, the researchers sequenced the complete coding region of the remaining wild-type allele and showed it was structurally intact. To exclude the possibility of downregulation or inactivation at the level of protein expression, they irradiated tumour-bearing mice prior to sacrifice, a treatment known to increase p53 protein levels via posttranslational mechanisms. Their data showed the retained wild-type allele in these tumours was expressed normally and suggested it had a normal wild-type conformation.

Next, the authors did a rigorous test of different functions of the p53 protein. They first tested whether the tumours showed amplification of Mdm2. This protein, whose expression is regulated by p53, stimulates breakdown of p53, thereby forming a negative feedback mechanism that keeps p53 levels low. Some tumours therefore amplify the *Mdm2* gene as a means of inactivating p53. However, this was not found in the tumours from the *Trp53* ^+/−^ mice. Subsequently, the researchers tested to what extent the retained *Trp53* copy behaved normally. Irradiation of many tissues leads to p53-dependent apoptosis, and, indeed, in tumours that had retained the wild-type allele, irradiation did lead to an increase in apoptosis, whereas in tumours that had lost the wild-type allele, it did not.

The p53 protein is known to function as a transcriptional regulator by either up- or down regulating target genes in response to different forms of cellular stress, including irradiation-induced DNA damage. The authors studied the expression of two p53-upregulated genes (*Cdkn1a*, which encodes p21, and *Mdm2*) and one downregulated gene (*Pcna*) in p53-positive tumours after irradiation and showed responses indicative of normal p53 function. Furthermore, it was shown that the p53 protein from the tumours was able to bind to a p53-binding DNA sequence in an in vitro setting. Finally, since it is known that p53 absence in tumours is correlated with chromosomal instability, Venkatachalam et al. (1998) used comparative genome hybridisation to compare this feature between p53-negative and p53-positive tumours and found a 5-fold greater stability in the latter.

In short, this paper clearly showed that, at least in mice, in many *Trp53* ^+/−^ tumours the wild-type allele of *Trp53* is not only retained, but also appears to function normally. This strongly suggested that a decrease of dosage in p53 is already sufficient for tumourigenesis, a phenomenon referred to as haploinsufficiency. Shortly before, the group of Moshe Oren (Gottlieb et al. 1997) had shown that a *Trp53* ^+/−^ background leads to a greater than 50% reduction in p53 activity using a p53-responsive *lacZ* reporter gene in transgenic mice. Venkatachalam and colleagues suggested the strong concentration dependence of p53 function could be explained by the fact that p53 functions as a tetramer. A 50% decrease in p53 monomers can easily be imagined to result in a greater than 50% decrease in functional tetramers, which in turn increases the chances of these cells becoming cancerous.

This paper by Venkatachalam et al. (1998) made me realise how important it is to remain critical, even of long-established theories and models. Since then, haploinsufficient mechanisms have been described in more tumour suppressor genes in humans and mice (reviewed in Fodde and Smits 2002). For instance, in a recent paper in *PLoS Biology*, Trotman et al. (2003) used mouse models to describe how the dosage of the *Pten* tumour suppressor gene influences the occurrence of prostate cancer. Further genes have been described with other unexpected roles in the tumourigenic process. There is a long-standing debate in the literature about the number and role of mutations in a tumour, and without going into the details, it is clear that haploinsufficient mechanisms for tumour suppressor genes will greatly influence the statistics on which these discussions are based. At a time when microarray analysis has become a standard experiment and the many thousands of changes in tumour cells are analysed across the whole genome, it is important to keep in mind that the correct interpretation of this wealth of information might be more complicated than the widely accepted models would have us believe.

## 

**Figure 1:**
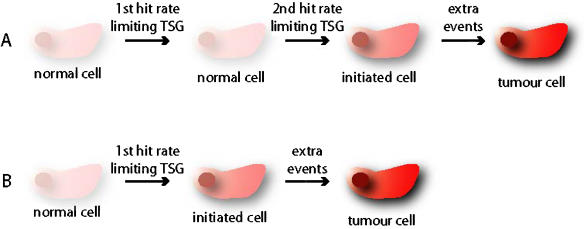
Initiating Genetic Aberrations in Tumourigenesis (A) According to the two-hit model, the first hit at the rate-limiting tumour suppressor gene provides no selective advantage for the cell. Only after the loss of the second allele of this gene is tumour formation initiated. Extra genetic changes are needed for complete transformation of the cell. (B) In a haploinsufficient mechanism, the first hit on the rate-limiting tumour suppressor gene already provides the cell with sufficient selective advantage to initiate tumour formation. Further events are necessary for complete transformation. These events might or might not include the loss of the wild-type allele of the rate-limiting tumour suppressor gene.
